# Role of the immune system in amyotrophic lateral sclerosis. Analysis of the natural killer cells and other circulating lymphocytes in a cohort of ALS patients

**DOI:** 10.1186/s12883-023-03255-x

**Published:** 2023-06-09

**Authors:** Tommaso Piccoli, Francesca Castro, Vincenzo La Bella, Serena Meraviglia, Marta Di Simone, Giuseppe Salemi, Francesco Dieli, Rossella Spataro

**Affiliations:** 1 Cognitive and Memory Disorders Clinic, AOUP “Paolo Giaccone” University Teaching Hospital and BiND, University of Palermo, Palermo, Italy; 2 ALS Clinical Research Center, Laboratory of Neurochemistry, AOUP “Paolo Giaccone” University Teaching Hospital and BiND, University of Palermo, Palermo, Italy; 3 Central Laboratory of Advanced Diagnosis and Biomedical Research, AOUP “Paolo Giaccone” University Teaching Hospital and BiND, University of Palermo, Palermo, Italy; 4 Multiple Sclerosis Clinic, AOUP “Paolo Giaccone” University Teaching Hospital and BiND, University of Palermo, Palermo, Italy; 5 ALS Clinical Research Center, Laboratory of Neurochemistry, Department of Biomedicine, Neurosciences and Advanced Diagnosis, University of Palermo, via Gaetano La Loggia, 1, Palermo, I-90129 Italy

**Keywords:** Amyotrophic Lateral Sclerosis, Circulating blood lymphocytes, NK lymphocytes, Disease progression, Biomarker

## Abstract

**Aims:**

Neuroinflammation might be involved in the degeneration and progression of Amyotrophic Lateral Sclerosis (ALS). Here, we studied the role of the circulating lymphocytes in ALS, in particular the NK cells. We focused on the relationship between blood lymphocytes, ALS clinical subtype and disease severity.

**Subjects and Methods:**

Blood samples were collected from 92 patients with sporadic ALS, 21 patients with Primary Lateral Sclerosis (PLS) and 37 patients affected by primary progressive multiple sclerosis (PPMS) with inactive plaques. Blood was taken from ALS and controls at the time of diagnosis/referral. Circulating lymphocytes were analyzed by flow cytometry with specific antibodies. Values were expressed as absolute number (n°/µl) of viable lymphocytes subpopulations in ALS were compared with controls. Multivariable analysis was made using site of onset, gender changes in ALSFRS-R and disease progression rate (calculated as ΔFS score).

**Results:**

Age at onset was 65y (58–71) in ALS (spinal 67.4%; bulbar, 32.6%), 57y (48–78) in PLS and 56y (44–68) PPMS. Absolute blood levels of the lymphocytes in the different cohorts were within normal range. Furthermore, while levels of lymphocytes T and B were not different between disease groups, NK cells were increased in the ALS cohort (ALS = 236 [158–360] vs. Controls = 174[113–240], p < 0.001). In ALS, blood levels of NK cells were not related with the main clinical-demographic variables, including the rate of disease progression. Multivariable analysis suggested that male gender and bulbar onset were independently associated with a risk of high blood NK cells levels.

**Conclusions:**

We show that blood NK cells are selectively increased in ALS, though their level appear unaffected in patients with an estimated rapidly progressing disease. Being of a male gender and with a bulbar onset seems to confer higher susceptibility to have increased NK lymphocytes levels at diagnosis/referral. Our experiments provides a further clear-cut evidence of the role of the NK lymphocytes as a significant player in ALS pathogenesis.

**Supplementary Information:**

The online version contains supplementary material available at 10.1186/s12883-023-03255-x.

## Introduction

Recent evidences suggest that neuroinflammation [NI] might be involved in the pathogenic pathways of several neurodegenerative disorders [1–7]. Amyotrophic Lateral Sclerosis (ALS) is the most common degenerative motoneuron (MN) disease, leading to rapidly progressive muscle weakness and death, mostly due to respiratory failure [8–11].

Pathogenic mechanisms underlying ALS are not fully understood and a number of factors seem to be involved, e.g. oxidative stress, protein misfolding, mitochondrial failure and neuroinflammation [8].

NI is increasingly documented as a key player in the ALS pathogenesis and as an important factor for disease progression [6]. Furthermore, the immune system plays either a protective role in the early stages of the disease through the activity of regulatory T cells (T-regs), T-helper 2 and M2 macrophages/microglia or a toxic effect in the late stages, through the involvement of the M1 macrophages/microglia and the pro-inflammatory T cells and a decrease of T-regs activity [6, 12–14]. For instance, the MN damage due to the accumulation of unfolded proteins, e.g., the mutant SOD1, induces a release of neurotoxic signals through a stressed endoplasmic reticulum: the consequent microglia activation leads to the release of pro-inflammatory cytokines and ROS [15]. Transgenic SOD1 mice, exposed to a combined treatment with interleukin-1/interleukin-2 monoclonal antibodies and rapamycin showed a restored number of Tregs and a prolonged survival.

Natural Killer cells (NKs) were found in the spinal cord and motor cortex of ALS patients as well as in SOD1 mice where showed an interaction with motor neurons and modulate immune response against motor neuron [16]. NKs infiltrate damaged CNS regions, attracted by the chemokine CCL2. The depletion of NKs cells decreases microglia activation by increasing Treg cells number and ameliorating survival in SOD1 and TDP43 mice [16]. Moreover, NKs modulate the cross-talk between Tregs and microglia, contributing to the onset of the disease. The role of NKs in ALS progression is, however, matter of debate [16, 17].

Recently, our group reported a case of very rapidly progressive motorneuron disease associated with NK leukemia showing clinical improvement after chemotherapy and consequent reduction in circulating NKs [18].

This work aimed to study the role of the immune system in a cohort of sporadic ALS patients, by measuring blood levels of T cells, CD4 + and CD8 + cells, B cells and NK cells in relationship with demographic and clinical variables, and to the disease progression as well.

## Materials and methods

### Patients

Between June 2019 and June 2022, we enrolled ALS consecutive 92 patients [M/F = 1.24], with a probable or a defined diagnosis of ALS according to El Escorial Revised criteria [19]. Disease controls were 58 patients [M/F = 1.46], of which 21 were affected by Primary Lateral Sclerosis [PLS] and 37 by Primary Progressive Multiple Sclerosis [PPMS], the latter with no MRI evidence of disease activity. ALS and PLS patients were recruited from the Tertiary ALS Clinical Research Center, Department of Biomedicine, Neuroscience and advanced Diagnostics, University of Palermo, Italy. PPMS patients were recruited from the Multiple Sclerosis Clinic from the same Department.

We scored the severity of ALS through the revised ALS Functional Rating Scale (ALSFRS-R) [20] and used the ∆FS ([ALSFRS-R at onset–ALSFRS-R at time of diagnosis]/diagnostic delay) to calculate the rate of progression (RoP) of the disease [21]. We then classified the patients into three groups based on their RoP: slow (∆FS < 0.5), intermediate (∆FS ≥ 0.5 < 1), rapid (∆FS ≥ 1). All patients gave informed written consent and underwent a blood withdrawal during their diagnostic workup or at referral. Therefore none of the patients was yet taking Riluzole. ALS patient’s referral is made to our Tertiary Center within one-three months from diagnosis (Spataro R, *unpublished data from the ALS Clinical Research Center*). PLS and PPMS patients were not at first referral/diagnosis.

### Flow cytometry of the circulating lymphocytes

Blood drawing was performed in fasted patients between 8:00 and 9:00 a.m. As the ALS patients and controls were free from immunosuppressive/immunomodulatory drugs, anti-inflammatory drugs, antibiotics, antiviral drugs and steroids since at least three months. All samples were kept at room temperature until analysis, which was done within two hours after blood withdrawal.

Circulating lymphocytes were analyzed by flow cytometry on a FACSC-anto II Flow Cytometer, upon staining cells with the following antibodies: antiCD3-FITC, antiCD16, antiCD56-PE, antiCD45-PerCpCy5.5, antiCD19-PeCy7, antiCD8-APC and antiCD4-APCH7. For each sample, values were expressed as both percent and absolute number (n/µl) of viable lymphocytes. Reference values for the adult healthy population were taken from Comans-Bitter et al.,1997 [22].

### Data analysis and statistics

All analyses were carried out using SIGMASTAT software 8.0 (Systat Software Inc., San Jose, CA, USA). Variables were expressed as median, with interquartile intervals (IQR). Both percent and absolute values of lymphomonocyte subpopulations (T lymphocyte CD3+, CD3+/CD4 + T, CD3+/CD8 + T, CD19 + B cells and CD3-/CD56 + NK cells) in ALS were compared with those subjects with PLS and/or PPMS. The non-parametric data were analyzed with the Mann-Whitney rank sum test. Categorial data were evaluated with the chi-square test. To compare data of multiple groups, we adopted the Kruskal - Wallis ANOVA on ranks. The Spearman correlation was used to assess the relationship between disease stage (i.e., ALSFRS-R score) and disease progression (ΔFS) with NK cells level. Multivariate logistic regression analysis was performed to assess certain variables independently associated with the circulating levels of the NK lymphocytes, as dependent variable. We therefore used the median number of NK cells/µl as cut-off value for a dichotomous outcome. The variables included in the analyses were as follows: age at onset, site of disease onset (S/B), gender (M/F), diagnostic delay, ΔFS. Moreover, a multiple regression was done to verify whether age was influencing the results. For all analyses, *p*-values < 0.05 were considered significant.

## Results

### Sample description

The demographic and clinical characteristics of the ALS cohort and the Disease Controls (DC) are summarized in Table 1. DCs were younger than the ALS at disease onset (median age: ALS, 65 years, IQR = 58–71, vs. DCs, 54 years, IQR = 40–68; *p* < 0.001, Mann-Whitney rank-sum test). The two groups were not different as M/F ratio, whereas the onset-to-assay interval, expressed in months, was obviously significantly shorter in the ALS (ALS = 15 months, IQR = 9–29 vs. DC = 47 months, IQR = 21–67; *p* < 0.001, Mann-Whitney Rank Sum Test). Among ALS patients, the Spinal/Bulbar ratio was 2.06. Other variables at the time of the assay were unique to ALS (i.e., ALSFRS-R, ΔFS and seated Forced Vital Capacity, FVC%) and define, as whole cohort, moderately disabled patients, with an intermediate rate of progression and an acceptable, though reduced below normal, seated FVC%. When DCs were divided into the two groups of PLS and PPMS, the relationship with the ALS cohort did not change (PLS and PPMS at onset were younger than ALS, but with a longer onset-to-assay interval (Suppl. TAB I). As expected, M/F ratio was higher than 1 in PLS and lower than 1 in PPMS.

**Table 1:** Clinical and Demographic characteristics of the ALS patients (n = 92) and the Disease Controls (DC: PLS + PPMS; n = 58). Data are expressed as median with interquartile ranges

Variable	ALS (n = 92)	DC (n = 58)	*p*
Age at onset	65 (58–71)	54 (40–68)	0.001*
Sex (M/F)	1.24	1.46	0.460**
Interval onset-assay (months)	15 (9–29)	47 (21–67)	< 0.001*
Onset			
Spinal, n (%)	62 (67.4)	n.a.	
Bulbar, n (%)	30 (32.6)	n.a.	
ALSFRS-R^a^	37 (32–42)	n.a.	
ΔFS	0.67 (0.38–1.17)	n.a.	
FVC%^a^	86 (67–95)	n.a.	

^a^evaluation at the time of the assay. * Mann-Whitney Rank Sum Test; ** chi-square; n.a.: not applicable

### Blood levels of circulating lymphocytes

Table 2 shows that the absolute blood levels of the different lymphocyte populations where within normal range [22]. However, while the CD3+, CD4+, CD8 + T-lymphocytes and the B-lymphocytes did not differ between ALS and DC, in the ALS patients the blood levels of the NK cells were significantly higher than in the controls (ALS, 236 cells/µl, IQR = 158–360, vs. DC, 174 cells/µl, IQR = 113–240, *p* < 0.001, Mann-Whitney Rank Sum Test).

**Table 2:** Levels (n° cells/µl) of different lymphocyte (Lymph) subtypes in ALS (n = 92) and disease controls (DC, n = 58). Data are expressed as median with interquartile ranges (IQR)

cell type (*n°* cells/µl)	ALS (n = 92)	DC (n = 58)	*p*
CD3 + T Lymph	1142 (861–1554)	1253 (1034–1628)	0.26
CD4 + T Lymph	773 (527–927)	799 (580–1192)	0.11
CD8 + T Lymph	398 (262–600)	418 (274–497)	0.70
B Lymph	177 (113–272)	191 (140–264)	0.21
NK cells	236 (158–360)	174 (113–240)	< 0.001

*p*, Mann-Whitney Rank Sum Test

### The NK lymphocytes in ALS

**Fig. 1:**
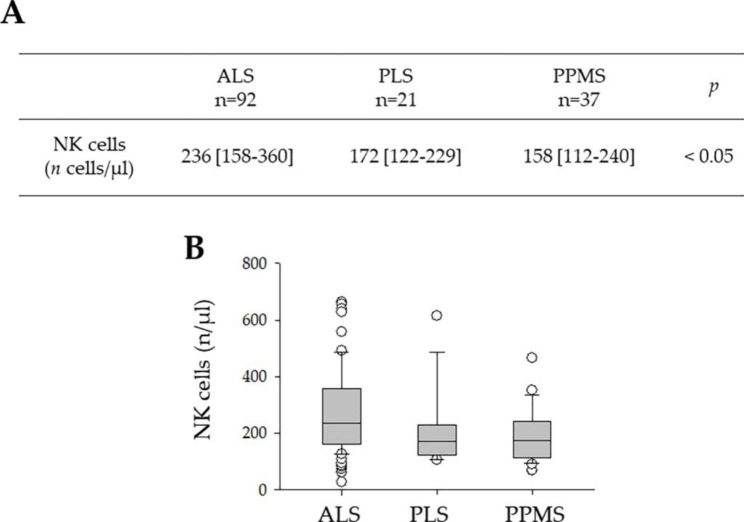
(**A**). Blood levels of NK cells in ALS (n = 92), PLS (n = 21) and PPMS(n = 37). Data are expressed as median with IQR. *p*, Kruskal-Wallis One-Way ANOVA on Ranks with a post-hoc Dunn’s analysis. (**B**) Histogram showing the different levels of the NK cells in the three groups (ALS, PLS and PPMS)

The finding that NK cells blood levels in ALS are higher than in the DCs, prompted us to evaluate variables that might be related to changes of this lymphocyte subpopulation. NK cells levels remained higher in ALS when compared to the two individual subgroups of Disease Controls (i.e., PLS and PPMS; Fig. 1A, B).

**Fig. 2:**
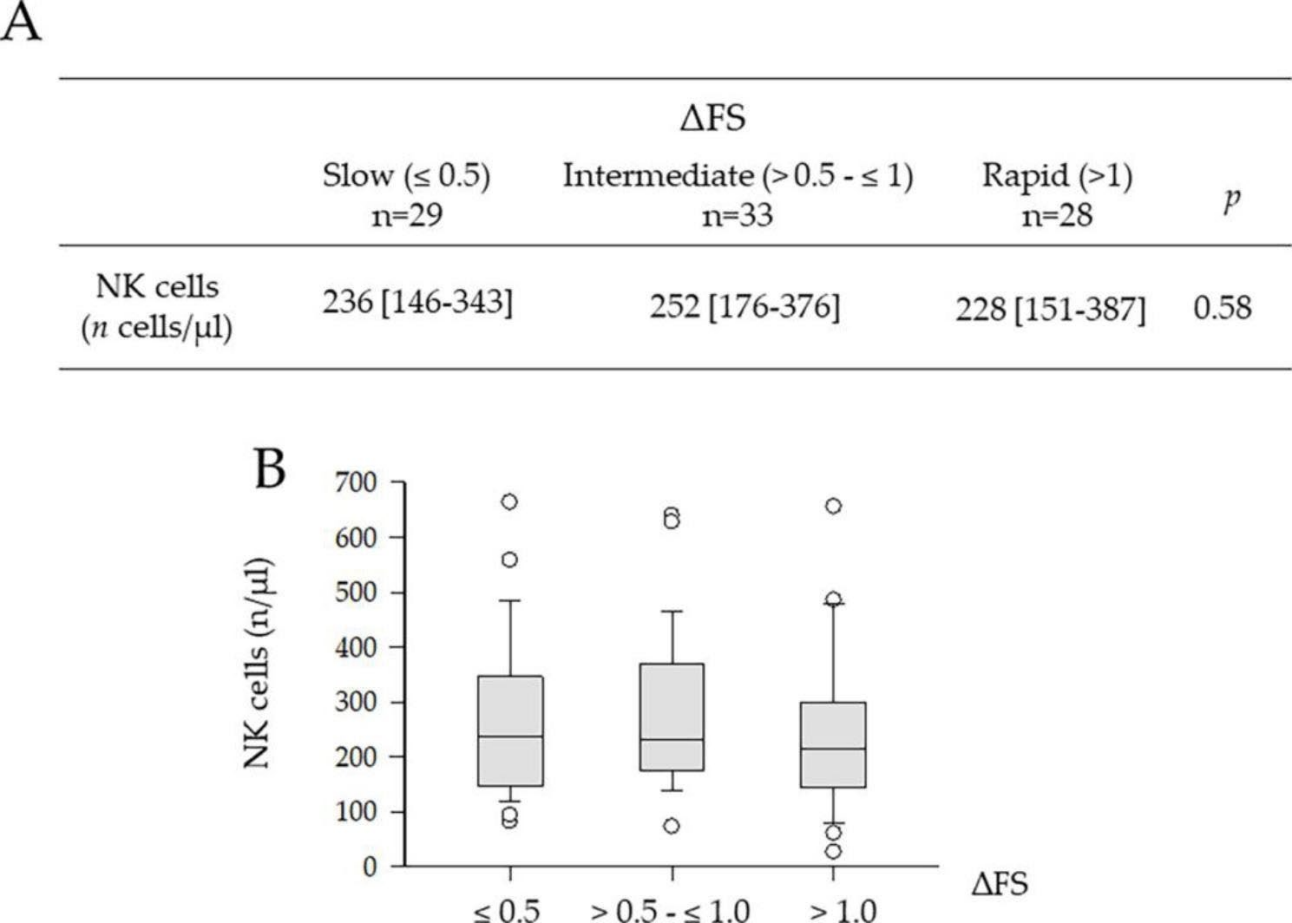
(**A**). Blood levels of NK cells in relationship to the rate of disease progression in ALS, as measured by ΔFS. Data are expressed as median with IQR. *p*, Kruskal-Wallis One-Way ANOVA on Ranks. (**B**). Histogram showing the levels of the NK cells in ALS patients according to the estimated rate of progression (ΔFS): slow [≤ 0.5]; intermediate [> 0.5 - ≤ 1.0]; rapid [> 1.0]

Disease progression was established with the ΔFS [21], and the ALS population was divided into three subgroups according to the rate of progression, i.e., slow, intermediate and rapid. Figure 2 A, B shows how the NK lymphocyte numbers were not significantly different across classes of disease progressors (Slow, 236 cells/µl [IQR = 146–343] vs. Intermediate, 252 cells/µl [IQR = 176–376] vs. 228 cells/µl [IQR = 151–387], *p* = 0.58, Kruskal-Wallis One-Way ANOVA on Ranks). These results were further supported the lack of relationship between DFS and NK cell levels by Spearman correlation analysis (*r* = − 0.10; *p* = 0.40; Suppl. Figure 1). We therefore suggest that NK cells level and the rate of progression are likely to be unrelated in this ALS cohort.

We then analyzed the relationship between the disease stage by ALSFRS-R and NK cells level. Again, we did not find a significant correlation between the two variables (*r* = -0.03; *p* = 0.77) by the Spearman correlation analysis (Suppl. Figure 2). Furthermore, a multiple regression analysis demonstrated that there was no significant relationship between age and any of the lymphocytes analyzed (analysis of variance, DF = 5, p = 0.11).

A multivariable logistic regression analysis was finally performed to investigate the independent association of demographic and clinical variables with the blood levels of the NK lymphocytes. As shown in Table 3, of the different variables explored (i.e., age at onset, gender, interval onset-to-assay, ALSFRS-R at the evaluation, ΔFS, FVC%, site of onset), the male gender and the bulbar site of onset were significantly associated with the NK cells levels (Gender, Male vs. Female, Odds ratio [OR] 5.08, CI = 1.61–20.9, p = 0.007; Site of onset, Spinal vs. Bulbar, OR 0.20, CI = 0.05–0.87, p = 0.033).

**Table 3:** Multivariate logistic regression analysis of variables associated with the blood level of ***NK cells*** (above median *vs* below median) in the ALS cohort.

Variable	odds ratio	95% CI	*p*
Reference:			
**NK cells** (≥ 236/µl)	1.0		
Age at onset (years)	0.94	0.89–1.00	0.092
Gender (M vs F)	5.08	1.61–20.9	**0.007**
Interval onset-assay (months)	1.00	0.96–1.05	0.748
ΔFS	0.88	0.30–2.54	0.82
ALSFRS-R at evaluation	0.97	0.89–1.05	0.49
FVC%	1.00	0.98–1.03	0.53
Site of onset (S vs B)	0.20	0.05–0.87	**0.033**

CI, confidence interval

## Discussion

Our results showed that blood levels of NK lymphocytes at diagnosis or at first referral is selectively and significantly elevated in ALS patients as compared to disease controls. Conversely, T cells (CD3+, CD4 + and CD8+) and B cells showed no difference. We found no correlation with the site of onset, disease duration, ALSFRS-R or the predicted rate of progression. Finally, male gender and bulbar site of onset were the only variables independently associated with higher blood levels of NK lymphocytes.

NKs are cytotoxic lymphocyte belonging to the innate immune system, able to recognize and attack cells infected by viruses, cancer cells as well as stressed and impaired cells such as neurons during neurodegeneration. When activated, NK cells secrete several cytokines such as interferon (IFN)-γ, TNF-α, the Granulocyte-Macrophage Colony-Stimulating Factor, several cytokines, that can act on other cells of the innate and acquired immunity, modulating their activity [23].

The NKs have been investigated in several neurological disorders with conflicting results. These cells may act as either protective or toxic agents in different diseases and even in different stages of the same disease [1, 3, 24, 25]. In particular, NK cells seem to have neurotoxic activity through activation of cytotoxic T cells and dendritic cells via the secretion of IFN-γ, but also through the release of perforin and granzyme B.

NK cells have also been extensively studied in animal models of neurological disorders.

In murine models of multiple sclerosis, NK cells seem to have a protective role, inducing a reduction in the neurotoxicity of self-reactive T cells by killing proinflammatory microglia [3]. Conversely, in the mouse model of ALS, expressing the human mutant G93A SOD1, NK cells accumulate in the motor cortex and spinal cord, determining MN degeneration by activating microglia and inducing a proinflammatory phenotype [16].

The increased circulating NK lymphocytes levels, we detected in the present study, are consistent with previous findings [26–28]. Murdok BJ et al. (2017) reported one longitudinal study on ALS patients [27]. The authors showed that levels of total leukocytes, neutrophils, and NK cells increase of over time, leading to the suggestion of a specific role of such cells in the disease progression. Rentzos M et al. (2012) found a higher number of CD8 T cells and NK in patients with ALS and a lower number of Tregs, the latter correlating with disease progression [26]. Finally, Gustafson MP et al. (2017) identified two different immune phenotypes in patients with ALS, one of which had a different pattern of blood cells than the controls, with early onset and slow progression. The authors also found a higher number of T cells including the NK [28].

We did not find any difference between ALS patients and controls in the levels of B lymphocytes and in the CD3+, CD4 and CD8 T-lymphocytes. This discrepancy could be explained by differences between selected ALS cohorts, especially in terms of age at onset and severity of the disease.

Our result of a relatively early high NK lymphocyte levels in ALS at diagnosis/first referral indicates that an immune involvement in the disease is an early likely-pathogenic event. We found that NK cells in our ALS cohort are not related with the predicted rate of progression (ΔFS). Now, we are evaluating the blood levels of this lymphocyte population longitudinally and in an ALS subpopulation carrying pathological expansions of C9orf72 G_4_C_2_ sextuplets (Spataro R et al., in preparation). Normal C9orf72 is acting by suppressing inflammation [29, 30]. Therefore, pathological expansions of C9orf72 lead to reduced levels of the protein, causing haploinsufficiency that cannot suppress inflammation. This mechanism is likely giving an appreciable contribution to ALS/ALS-FTD pathogenesis [29, 30].

NK cells are able to infiltrate brain and spinal cord of ALS animal models and humans [16] and seem to exert a direct toxic effect against motoneurons [16, 31]. We analyzed both ΔFS (a predictor of rate of progression) and the NK lymphocyte levels when the disease was in an early stage. Therefore, the lack of difference in NK levels between the three different rates of progression is not surprising. Nevertheless, we postulate that during the disease evolution, blood NK levels should decrease in the blood, as these lymphocytes proceed to infiltrate the nervous tissue [16].

Another interesting result of our work was the difference of circulating NK cells levels between sexes. There is now large amount of evidences supporting a role of sex as risk factor for many neurological conditions, with different impact on epidemiology and pathogenic mechanisms [11, 32–36]. Recently, Murdock BJ et al. (2021) found a specific sex effect after NKs reduction in transgenic mice, with a positive clinical response for survival in females, though in the same article they found no difference in the total number of blood NKs in patients with ALS, when stratified for sex [31].

Finally, our description of a paraneoplastic aggressive ALS in a man with acute NK leukemia support the potential role of this class of lymphocytes in the disease pathogenesis [18].

In conclusion, our work is a further evidence of the involvement of the immune system in neurodegenerative disorders, underpinning the role of the NK lymphocytes as a major player in ALS, which could be gender-related.

ALS is a complex neurodegenerative disorder, in which many different factors (genetic, environmental, immune, etc.) play a role in its pathogenesis and progression [5, 37–40]. In this context, a central pathogenic role seems to be played by the NK lymphocytes, which also show the potential of being an attractive therapeutic target for the disease [5, 31].

## Electronic supplementary material

Below is the link to the electronic supplementary material.

Supplementary Material 1

## Data Availability

All collected data are available from the corresponding author upon reasonable request.
