# Supplementary material for: Role of the immune system in amyotrophic lateral sclerosis. Analysis of the natural killer cells and other circulating lymphocytes in a cohort of ALS patients
Source: BMC Neurol. 2023 Jun 9;23:222. doi: 10.1186/s12883-023-03255-x (PMC10251617; doi:10.1186/s12883-023-03255-x)
Supplement: Supplementary file 1 — Supplementary Material 1 [file 12883_2023_3255_MOESM1_ESM.docx]

***BMC Neurology***: Research paper

**ROLE OF THE IMMUNE SYSTEM IN AMYOTROPHIC LATERAL SCLEROSIS.**

**ANALYSIS OF THE NATURAL KILLER CELLS AND OTHER CIRCULATING LYMPHOCYTES IN A COHORT OF ALS PATIENTS**

Tommaso Piccoli^1^, Francesca Castro^2^, Vincenzo La Bella^2^*, Serena Meraviglia^3^, Marta Di Simone^3^, Giuseppe Salemi^4^, Francesco Dieli^3^, Rossella Spataro^2^

^1^ Cognitive and Memory Disorders Clinic; ^2^ ALS Clinical Research Center and Laboratory of Neurochemistry; ^3^ Central Laboratory of Advanced Diagnosis and Biomedical Research; ^4^ Multiple Sclerosis Clinic; AOUP “*Paolo Giaccone*” University Teaching Hospital and Department of Biomedicine, Neurosciences and Advanced Diagnosis, University of Palermo, 90129, Palermo, Italy.

**Supplemental Material R1**

**Suppl TAB I. Clinical and Demographic characteristics of the ALS patients and the Disease Controls, divided into two groups according to the disease type.**

**DC-1:** PLS; **DC-2**: PPMS. Data are expressed as median with interquartile ranges.

___________________________________________________________________________

**ALS** **DC-1 (PLS)** DC-2 (PPMS)

Variable (n=92) (n=21) (n=36) *p*

___________________________________________________________________________

Age at onset 65 (58-71) 57 (48-78) 56 (44-68) 0.01 *

Sex (M/F) 1.24 1.7 0.62 0.032 **

Interval onset-assay

(months) 15 (9-29) 54 (32-120) 38 (18-55) < 0.001 *

___________________________________________________________________________

* Kruskal-Wallis One Way Analysis of Variance on Ranks; ** chi-square;


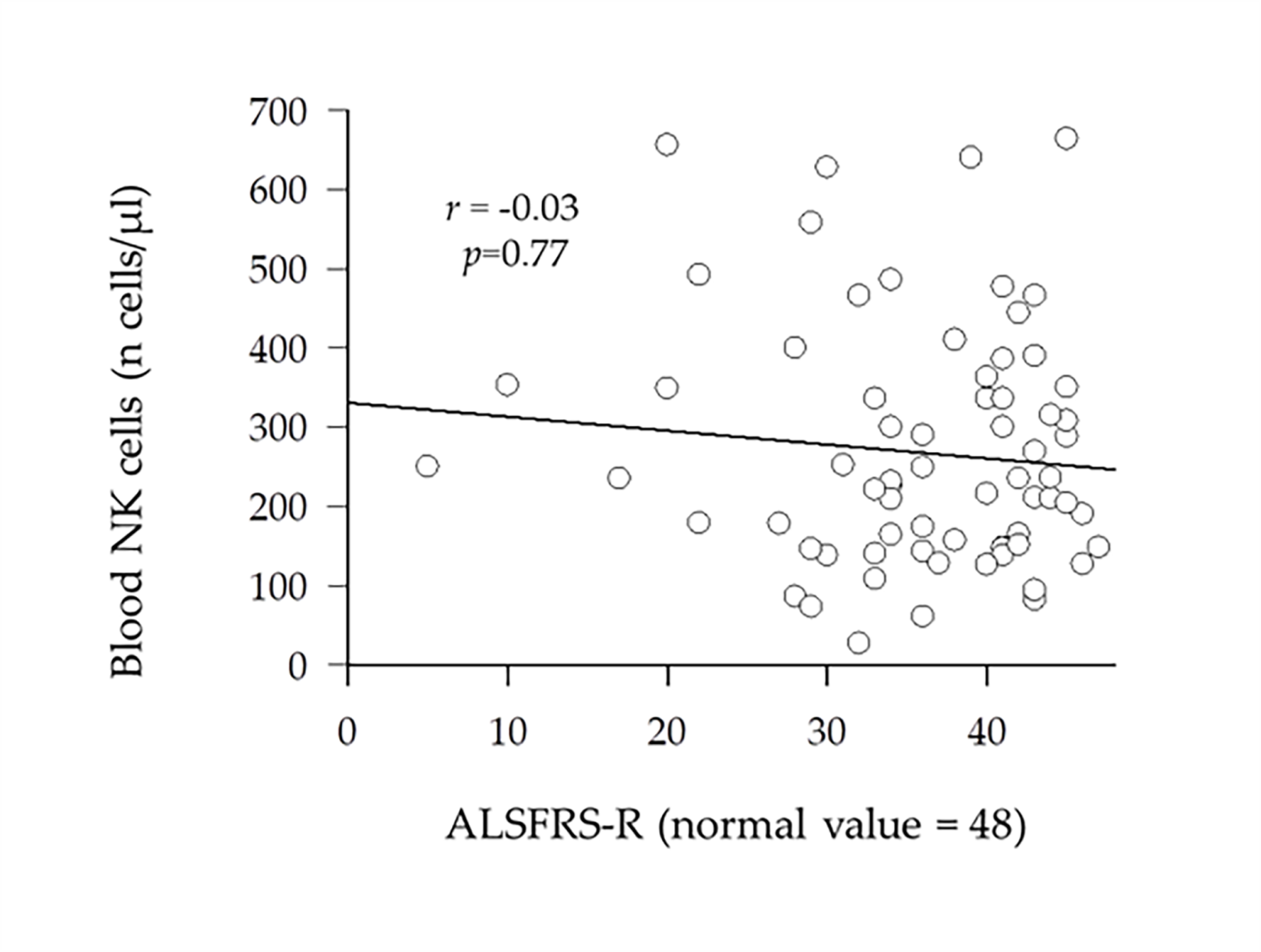


**Suppl FIG 1**. Relationship between circulating NK cells and ALSFRS-R at the time of blood drawing (diagnostic work-up or referral) in ALS patients made with the Spearman correlation analysis. No significant correlation was found between the two variables.


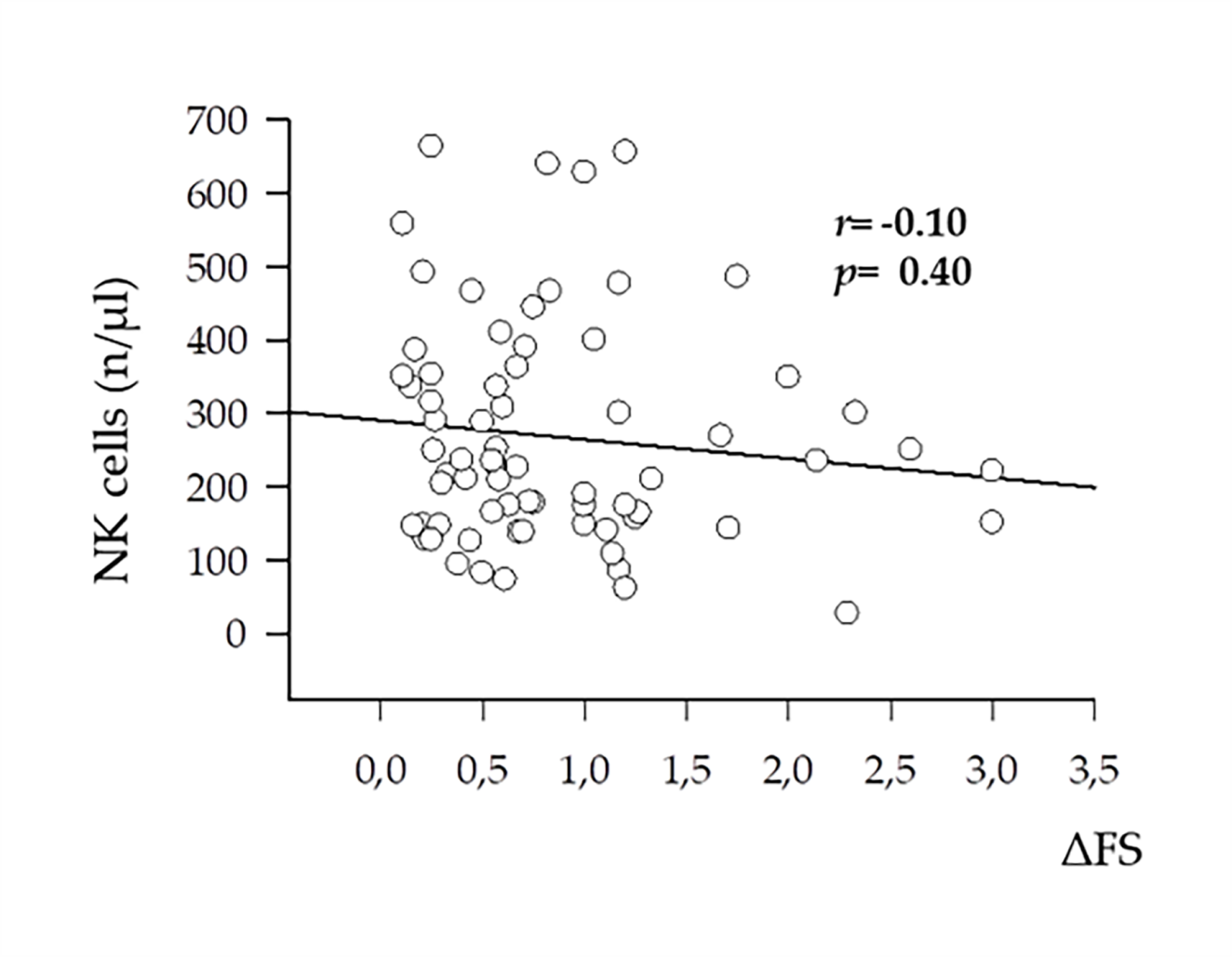


**Suppl FIG 2**. Relationship between circulating NK cells and ΔFS (a measure of disease progression) in ALS patients made with the Spearman correlation analysis. No significant correlation was found between the two variables.
